# Comparative Genomics Analysis of Two Different Virulent Bovine* Pasteurella multocida* Isolates

**DOI:** 10.1155/2016/4512493

**Published:** 2016-12-14

**Authors:** Huihui Du, Rendong Fang, Tingting Pan, Tian Li, Nengzhang Li, Qiang He, Rui Wu, Yuanyi Peng, Zeyang Zhou

**Affiliations:** ^1^The State Key Laboratory of Silkworm Genome Biology, Southwest University, Beibei, Chongqing 400716, China; ^2^College of Animal Science and Technology, Southwest University, Beibei, Chongqing 400716, China

## Abstract

The* Pasteurella multocida* capsular type A isolates can cause pneumonia and bovine respiratory disease (BRD). In this study, comparative genomics analysis was carried out to identify the virulence genes in two different virulent* P. multocida* capsular type A isolates (high virulent PmCQ2 and low virulent PmCQ6). The draft genome sequence of PmCQ2 is 2.32 Mbp and contains 2,002 protein-coding genes, 9 insertion sequence (IS) elements, and 1 prophage region. The draft genome sequence of PmCQ6 is 2.29 Mbp and contains 1,970 protein-coding genes, 2 IS elements, and 3 prophage regions. The genome alignment analysis revealed that the genome similarity between PmCQ2 and PmCQ6 is 99% with high colinearity. To identify the candidate genes responsible for virulence, the PmCQ2 and PmCQ6 were compared together with that of the published genomes of high virulent Pm36950 and PmHN06 and avirulent Pm3480 and Pm70 (capsular type F). Five genes and two insertion sequences are identified in high virulent strains but not in low virulent or avirulent strains. These results indicated that these genes or insertion sequences might be responsible for the virulence of* P. multocida*, providing prospective candidates for further studies on the pathogenesis and the host-pathogen interactions of* P. multocida*.

## 1. Introduction

*Pasteurella multocida *(*P. multocida*) is the etiologic agent of bovine pneumonia and hemorrhagic septicemia in cattle which has been estimated to cause huge economic losses. Five capsule types are routinely identified in* P. multocida *(A, B, D, E, and F) and each is generally associated with, but not completely restricted to, a specific host [1].* P. multocida *has the typical characteristics of an opportunistic pathogen that is affected by various host and pathogen specific determinants and can survive in the oral cavity and upper respiratory tract of wild and domestic animals. In both, animals and humans,* P. multocida *is often associated with chronic as well as acute infections that can lead to significant morbidity (manifested as pasteurellosis, pneumonia, atrophic rhinitis, hemorrhagic septicemia and/or cellulitis, abscesses, and meningitis) and mortality, particularly in animals [2, 3]. Nevertheless, pasteurellosis is still a relatively uncommon cause of mortality in human, even though deaths due to pasteurellosis have increased in recent years in the United States [4, 5], and pasteurellosis in human is often due to bites or scratches by cats or dogs [6, 7].

The first complete genome sequence of* P. multocida* was Pm70, isolated from avian species in 2001 [8]. Since then, the complete or incomplete genomes of 57* P. multocida* isolates have been sequenced, including at least ten complete genomes from the species in the NCBI database. All of the currently available* P. multocida* genomes are between 1.43 Mbp and 2.44 Mbp in length and comprise a single circular genome with a G+C content between 36.9% and 41%. The available data were used to identify a number of important similarities and differences between these strains and determine their virulence [9].

Several species-specific putative virulence factors, including the capsular and virulence-associated genes, have been proposed to play a key role in the interactions with the host [10, 11].* P. multocida* possesses a number of virulence factors which include polysaccharide capsule, endotoxins or lipopolysaccharide (LPS), outer membrane proteins (OMPs), fimbriae, exotoxins, multocidins or siderophores, extracellular enzymes, plasmids, and the virulence-associated genes (*tbpA*,* pfhA*,* toxA*,* hgbB*,* hgbA*,* Fur*,* tonB*,* exbB*,* hgbB*,* nanH*,* nanB*,* sodA*,* sodC*,* ompA*,* ompH*,* oma87*,* PlpB*,* fimA*,* hsf-1*,* hsf-2*,* tadD, *and* ptfA*) [1, 12, 13]. It is speculated that the virulence factors expressed by* P. multocida *are likely to play key roles in pathogenesis. Comparative genomics provides an effective source for better understanding the virulence of different isolated strains. In this study, genome sequencing and comparative genomics analysis were carried out to investigate the underlying virulence factors of the high virulent and low virulent bovine* P. multocida* capsular type A strains, PmCQ2 and PmCQ6, respectively.

## 2. Materials and Methods

### 2.1. Bacterial Strains and Culture Conditions

Two* P. multocida* isolates (PmCQ2 and PmCQ6) have been previously isolated from the fatal pneumonia lungs of feedlot calves at Gaojiazhen farms in Fengdu (Chongqing, China, longitude/latitude 107.70/29.89) from 2011 to 2012. Based on morphological characteristics, biochemical properties, and 16SrRNA gene sequence analysis, the bacteria were identified as* P. multocida*. Further analysis with PCR amplification of* P. multocida* species-specific gene* Kmt-1* and serotype-specific genes* hyaD-hyaC, bcbD, dcbF, ecbJ, *and* fcbD* [16] indicated that the isolates were* P. multocida *capsular type A, named as PmCQ2 and PmCQ6, and the virulence of the two strains determined by LD_50_ in Kunming mice showed that PmCQ2 is a high virulent strain and PmCQ6 is a low virulent strain with 2.2 × 10^5^ CFU and 1.14 × 10^8^ CFU, respectively [17]. Isolated strains were maintained at −80°C in Martin Broth (MB) plus 10% glycerol. PmCQ2 and PmCQ6 were inoculated in 5 mL MB at 37°C overnight with shaking. The concentration was determined by viable cell counting on Martin agar plates at 37°C for 24 h.

### 2.2. Genome Sequencing and Annotation

Genomic DNAs of the two strains were isolated using the Qiagen DNA extraction kits. Genome sequencing was performed using an Illumina MiSeq platform. A total of 6,394,560 and 525,022,200 paired-end 100 bp reads of each genome were assembled into 7 and 32 contigs for strains PmCQ2 and PmCQ6, respectively. The sequences of PmCQ2 and PmCQ6 were assembled by SOAPdenovo [18]. Assemblies were submitted to NCBI for analysis. Open reading frames (ORFs) were annotated by searching against the Nr, Swiss-Prot, and COG databases with manually curation using BLASTP (*e*-value < 1*e* − 5) (Table S1 in Supplementary Material available online at http://dx.doi.org/10.1155/2016/4512493). The rRNA and tRNA genes were identified using RNAmmer [19] and tRNAscan [20], respectively. A comprehensive genome map containing coding and noncoding genes, COG annotations, and overall G+C content was plotted using Perl-SVG [21].

### 2.3. Global Alignment Analysis

MUMmer is ideally suited for aligning genomes when the genome sequences are very similar and provides genome-wide sequence comparisons to determine the maximum unique matches between two sequences [22]. Here, MUMmer and BLASTN (*e*-value of 1*e* − 10) were applied for a detailed collinearity analysis of the three bovine Pm genomes, PmCQ2, PmCQ6, and Pm36950 at nucleotide sequence levels. Pm36950 is also bovine* P. multocida* capsular type A strain and was obtained from the NCBI Genebank and was used as the reference genome sequence.

### 2.4. BLAST Score Ratio Analysis

Genes that were unique to each strain were also identified using BLASTN. The BLAST score ratio (BSR) method was used to compare peptide identities within three genomes (PmCQ2, PmCQ6, and Pm36950) using a measure of similarity based on the ratio of BLAST scores. The output of the BSR analysis enables global visualization of the degree of proteome similarity among genomes and enables the genomic synteny (conserved gene order) between each genome pair to be assessed [23]. Pm36950 was used as a reference genome sequence. The BSR was calculated by dividing the query score by the reference score for each reference peptide. Following calculation of the BSRs, the four quadrants were derived from a BSR threshold value of 0.4, which was empirically determined to represent approximately 30% amino acid identity over approximately 30% of the peptide length and is a commonly used threshold for peptide similarity [24]. The four quadrants were determined for each of the query genomes and colored accordingly: yellow, unique to the reference, PmCQ2 < 0.4, and PmCQ6 < 0.4; red, common to all three, PmCQ2 ≥ 0.4, and PmCQ6 ≥ 0.4; Green, common between PmCQ2 and Pm36950, but absent in PmCQ6, PmCQ2 < 0.4, and PmCQ6 ≥ 0.4; Blue, common between PmCQ6 and Pm36950, but absent in PmCQ2, PmCQ2 ≥ 0.4, and PmCQ6 < 0.4.

### 2.5. Virulence Factors

Prophage-associated gene clusters were identified by PhiSpy [25]. Genomic islands (GIs) are clusters of genes in prokaryotic genomes of probable horizontal origin. GIs of* P. multocida* were predicted with IslandPick [26]. Insertion sequences (ISs) of* P. multocida* were identified by searching sequences against the IS Database (Table S1) that collects all ISs of bacteria and archaea. ISFinder [27] was implemented to launch BLAST with the *e*-value 1*e* − 10 to search the database. Membrane proteins generally include transmembrane domains and were predicted by TMHMM Server 2.0 [28]. Signal peptide, transmembrane domain, GPI-anchor, and general subcellular localization were predicted with SignalP v3.0 [29], TMHMM Server 2.0, GPI-SOM [30], and PSORTb [31] to screen potential secretory proteins that contain signal peptide and no membrane localization signals. The virulence factor database (VFDB) is an integrated and comprehensive online resource for curating information about virulence factors of bacterial pathogens (Table S1). Based on homologous analysis, some virulent factors (ISs, GIs, VF, secretory proteins, and membrane proteins) were obtained in the sequenced strains. In combination with the potential virulent genes of* P. multocida* and gene annotation information, putative virulence genes for each strain were presented.

## 3. Results

### 3.1. Overview of the* P. multocida* PmCQ2 and PmCQ6 Genomes

**Figure 1:**
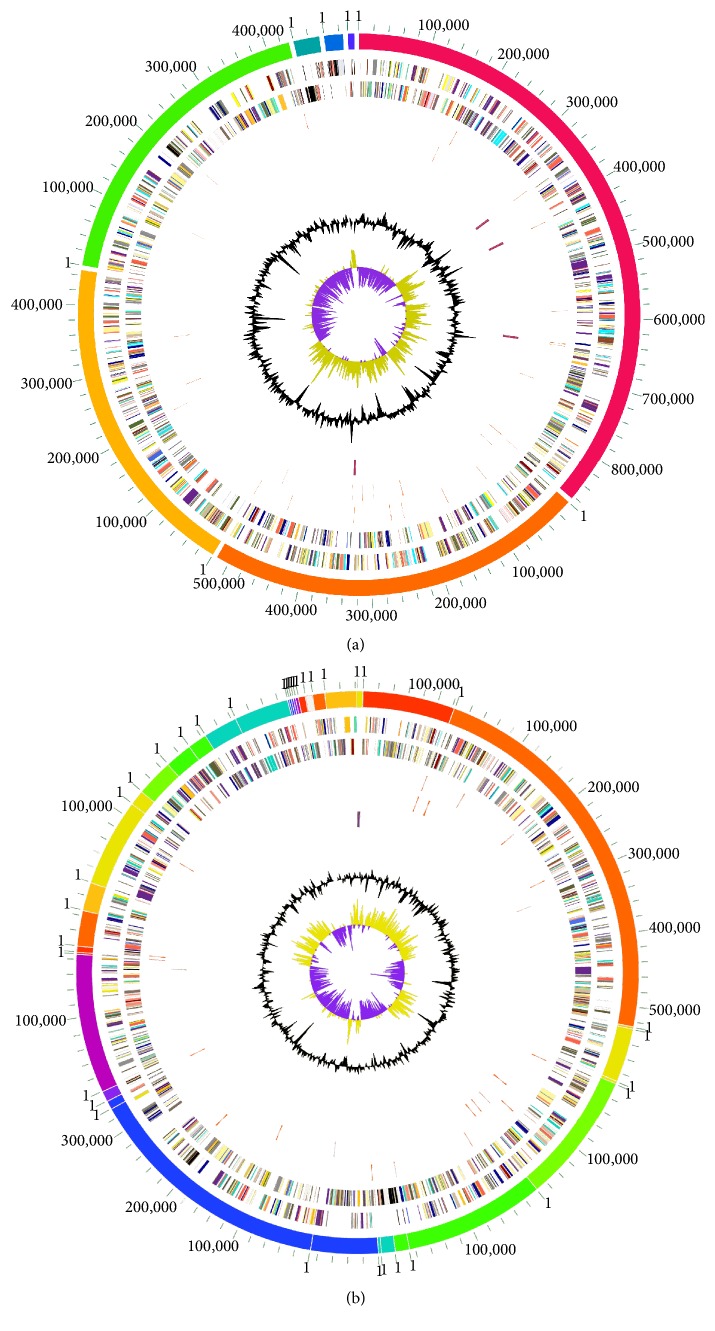
Circular genome maps of PmCQ2 (a) and PmCQ6 (b) from inside to outside indicate the following: Circle 1, G+C skew; yellow green, G+C skew > 0; purple, G+C skew < 0; Circle 2, G+C content (median represents the above average content, the outer circle is greater than the average content, and the inner circle is less than the average content); Circle 3, rRNA genes distribution represented in scaffold sequence; Circle 4, tRNA gene distribution represented in scaffold sequence; Circle 5, open reading frame (ORF) distribution, plus strand; and Circle 6, multiple scaffold exhibition.

The genome sequences of bothPmCQ2 and PmCQ6 strains were successively sequenced by Illumina MiSeq platform. Using Pm36950 as a reference strain, PmCQ2 genome is 2.32 Mbp in size with 39.12% G+C content, containing 2,000 predicted coding regions, 4 rRNAs operons, and 49 tRNAs. PmCQ6 genome is 2.29 Mbp in size with 40.09% G+C content, containing 1,969 predicted coding regions, 1 rRNA operon, and 43 tRNAs. The single circular genome maps of the two* P. multocida* genomes were shown in Figure 1. There are no obvious species-specific features of the coding density, and the G+C content is highly conserved. Compared with some other* P. multocida* strains carrying multiple plasmids that may either be cryptic or carry antibiotic resistance genes, both PmCQ2 and PmCQ6 genomes do not contain any plasmids. Taken together, there are only slightly differences in genome sizes, predicted gene numbers, and G+C contents between PmCQ2 and PmCQ6.

### 3.2. COG Classification

**Figure 2:**
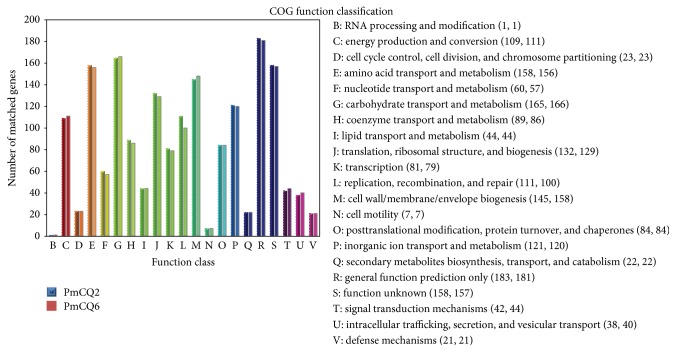
Clusters of Orthologous Group annotations for the genomes of PmCQ2 and PmCQ6. Arabic colon-separated numbers in brackets indicate matched proteins in PmCQ2 and PmCQ6.

The predicted protein sequences were annotated to various COG categories. Some differences in protein numbers among COG categories of PmCQ2 and PmCQ6 were identified (including those listed as protein numbers for PmCQ2 and PmCQ6, resp.): “energy production and conversion” (109 and 111), “amino acid transport and metabolism” (158 and 156), “nucleotide transport and metabolism” (60 and 57), “carbohydrate transport and metabolism” (165 and 166), “coenzyme transport and metabolism” (89 and 86), “translation, ribosomal structure, and biogenesis” (132 and 129), “transcription” (81 and 79), “replication, recombination, and repair” (111 and 100), “cell wall/membrane/envelope biogenesis” (145 and 158), “inorganic ion transport and metabolism” (121 and 120), “general function prediction only” (183 and 181), “function unknown” (158 and 157), “signal transduction mechanisms” (42 and 44), and “intracellular trafficking, secretion, and vesicular transport” (38 and 40) (Figure 2).

### 3.3. Global Alignment Analysis

**Figure 3:**
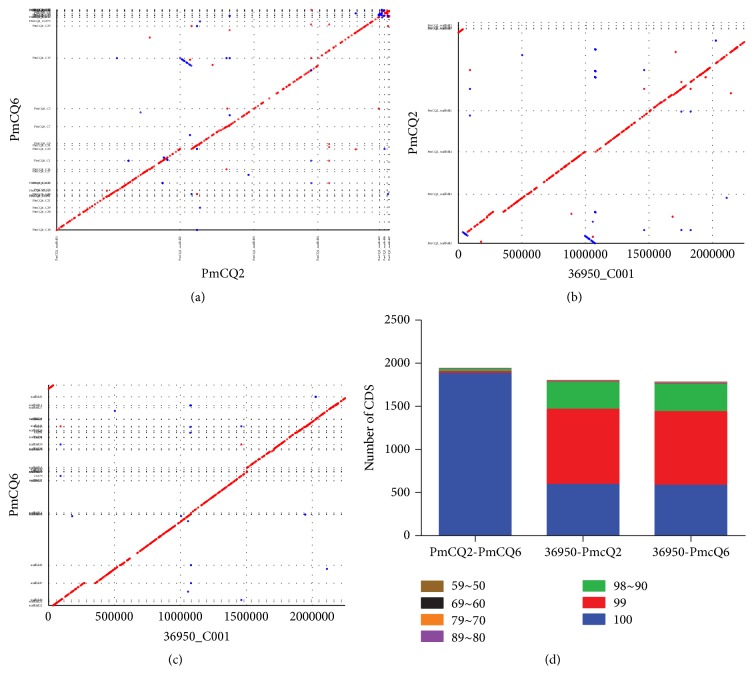
The global alignment analysis of three bovine Pm capsular type A genomes. Aligned segments are represented as dots or line. The alignment was generated by the mummerplot script and the Unix program gnuplot. (a) PmCQ2 and PmCQ6 genome sequences are given on the *x*- and *y*-axis, respectively. (b) Pm36950 and PmCQ2 genome sequences are given on the *x*- and *y*-axis, respectively. (c) Pm36950 and PmCQ6 genome sequences are given on the *x*- and *y*-axis, respectively. Dot plot indicted the alignment blocks of two genome alignment sequences; red and blue indicted the forward and the reverse sequence, respectively. (d) Direct comparison of the three nucleotide sequences using BLAST. The vertical coordinates are the number of genes. Percentage of genetic similarity is indicated by color coding.

**Figure 4:**
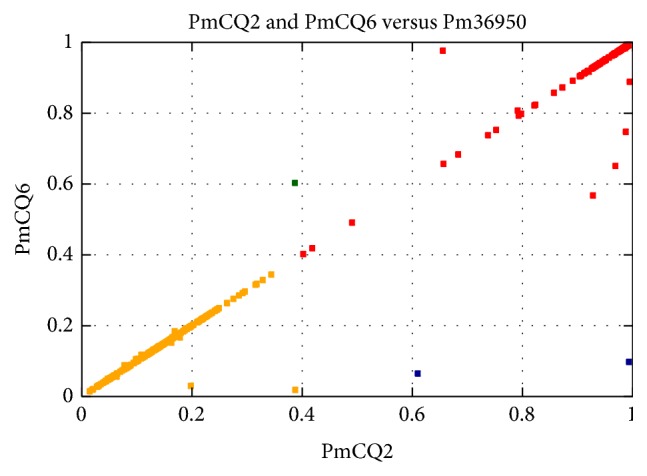
The distribution diagram of BLAST score ratio (BSR) between PmCQ2, PmCQ6, and Pm36950. Pm36950 was obtained from NCBI and used as a reference genome sequence. The color coding is as follows: yellow: PmCQ2 < 0.4 and PmCQ6 < 0.4; red: PmCQ2 ≥ 0.4 and PmCQ6 ≥ 0.4; green: PmCQ2 < 0.4 and PmCQ6 ≥ 0.4; blue: PmCQ2 ≥ 0.4 and PmCQ6 < 0.4.

**Figure 5:**
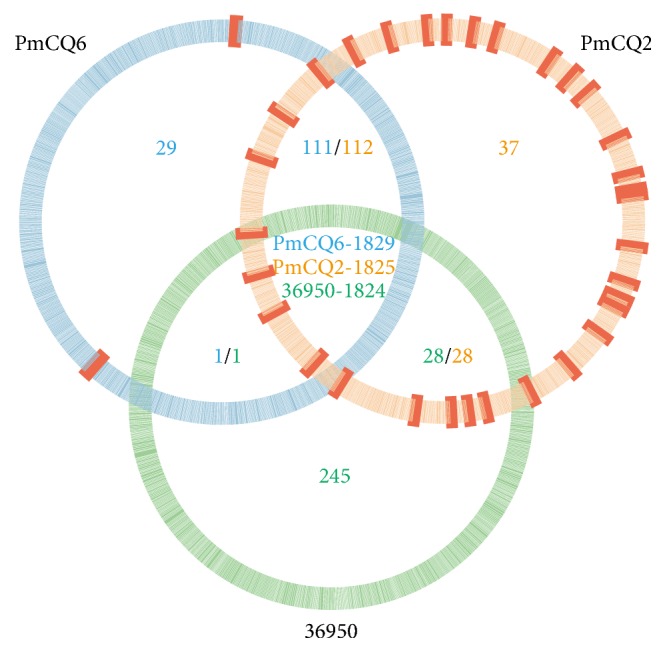
Venn diagram illustrating the number of putative proteins associated with each organism and the number shared with the intersecting organism. Red thick marks on each circle represent the location of the unique proteins (BLAST score ratio less than 0.4) on the PmCQ2 and PmCQ6 genome. Chromosomal comparison: jacinth, PmCQ2; blue, PmCQ6; green, Pm36950.

The colinearity analysis at the nucleotide level provides information on sequence insertion or deletion [32]. By aligning the genome at the nucleotide level, there was no significant differences among the large segments between high virulent PmCQ2 and low virulent PmCQ6, and the two strains revealed high colinearity with Pm36950 (Figures 3(a)–3(c)). Direct comparison of the complete nucleotide sequences using BLAST revealed the similarity between PmCQ2 and Pm36950, PmCQ6 and Pm36950, and PmCQ2 and PmCQ6 is 90%, 90%, and 99%, respectively. PmCQ2 and PmCQ6 showed higher homology as indicated by matched CDS (Figure 3(d)). By BSR analysis, the protein sequences shared a high degree of synteny among PmCQ2, PmCQ6, and Pm36950, using Pm36950 as a reference strain (Figure 4). However, some unique proteins were identified, PmCQ2 and PmCQ6 (BLAST score ratio is less than 0.4). There are 32 unique proteins in PmCQ2 genome (including transposase IS200, elongation factor Tu-A-1/2, SrfC, lsrR, TolA, and peptidase B) and only two unique proteins found in PmCQ6 genome (*Pasteurella* filamentous hemagglutinin protein and mercuric transport protein MerT). The relative chromosomal locations of the unique proteins (red thick marks) of PmCQ2 and PmCQ6 were shown in Figure 5.

Using a Venn diagram of three bovine* P. multocida *strains, the majority of homologous gene groups and unique gene groups were identified. The unique gene groups were significantly different among three strains, containing 37, 29, and 245 gene groups in PmCQ2, PmCQ6, and Pm36950, respectively (Figure 5).

### 3.4. Virulence Factors

The pathogenicity of* P. multocida* is associated with different virulence factors. The major virulence factors identified in* P. multocida* are capsule proteins, lipopolysaccharides, membrane proteins, and secreted proteins. Here, together with genome sequences of PmCQ2 and PmCQ6, published genome sequences of high virulent strains (Pm36950 and PmHN06) and avirulent strains (Pm3480 and Pm70) from NCBI were selected for comparative genomics analysis (Table 1). Comparing the PmCQ2 and PmCQ6 genomes with the complete genome sequences of Pm36950 (G+CA_000234745.1), PmHN06 (G+CA_000255915.1), Pm3480 (G+CA_000259545), and Pm70 (G+CA_000006825.1) using BLAST, a number of virulence-associated genes were identified that were absent or present in all of the comparison strains (Table 2).

A number of genes or gene clusters have been implicated as important for virulence of* P. multocida* [9]. Some of these genes encoding putative virulence factors are universally present in all six* P. multocida* genomes, including genes encoding prophage, genomic islands, insertion sequences, virulence factor, secretory proteins, and outer membrane proteins.

By comparing the high virulent strains (PmCQ2, Pm36950, and PmHN06) with low virulent strain (PmCQ6) and avirulent strains (Pm3480 and Pm70), unique genes which were correlated with virulence and only presented in high virulent strains were identified. For instance, insertion sequence (transposase IS200) only existed in three high virulent strains, suggesting that IS200 elements are not conserved sequences and do not spread among all* P. multocida* strains. IS605 and secreted protein PmCQ2_2g0088 (ModB) and nonspecific tight adherence protein D PmCQ2_3g0367 were presented only in PmCQ2 genome (Table 2).

In addition, genomic islands (GIs) are clusters of genes in prokaryotic genomes and are probable horizontal origin. GIs of Pm70, Pm3480, Pm36950, and PmHN06 were predicted with IslandPick. Homology analysis of these GIs with the draft genomes of PmCQ2 and PmCQ6 was carried out using ORTHOMCL1.4 (BLAST *p* value 1*e* − 5, percent identity cutoff 60%, and percent match cutoff 60%). The result showed that transcriptional regulator* PmCQ2_7g0006* and hypothetical proteins* PmCQ2_5g0013 *and* PmCQ2_5g0025 *are present in high virulent strains (PmCQ2 and PmHN06) but absent in low virulent strain PmCQ6 and the avirulent strains (Pm70 and Pm3480).

Taken together, comparative genomics analysis supplies essential information for understanding the virulence of different capsular type (A, D, and F) and different host origin (bovine, avian, and swine) strains. Five unique genes and two insertion sequences were identified only in high virulent strains, providing candidate virulence factors for further studies on the pathogenesis of different* P. multocida *strains (Table 3).

## 4. Discussion

Moreover, comparative genomic analysis allows the identification of core genes and/or disease-specific factors. The first complete* P. multocida* genome was sequenced from strain Pm70 in 2001, from which 104 putative virulence-associated genes were identified [8]; this facilitated new approaches for studying the pathogenesis of* P. multocida*. Until now, the complete and incomplete genomes of 57* P. multocida* have been sequenced in NCBI database. In this study, two bovine* P. multocida* capsular type A genomes (high virulent PmCQ2 and low virulent PmCQ6) were sequenced. Comparative genomics analysis was performed among PmCQ2, PmCQ6, and four other* P. multocida* genomes (Pm36950, PmHN06, Pm3480, and Pm70) from NCBI. Some virulence genes were identified among different virulent strains; five genes and two insertion sequences were only identified in high virulent strains, which might be responsible for the virulence differences among high virulent, low virulent, and avirulent strains.

The genome sequences of high virulent PmCQ2 and the low virulent PmCQ6 have high similarity, but the virulence of two strains is significantly different. It could be speculated that the unique genes may play a key role in virulence. Compared with PmCQ6, the five genes and two insertion sequences are predicted virulence-associated genes in PmCQ2 and other high virulent strains. Further studies to construct mutant strains targeting these genes would be of great importance to prove their contributions to virulence. Besides, PmCQ2 has more than 30 other unique genes that might also orchestrate the virulence differences of PmCQ2 and PmCQ6. These genes include recombinase, phage-related genes, phage N-6-adenine-methyltransferase, phage terminase, and prophage integrase.

Based on homology analysis, prophage-associated genes, GIs, ISs, secretory proteins, and membrane proteins were screened for different virulence-associated genes among different virulent strains. Insertion sequences usually only carry genes of transposon sequences for the transposition in bacteria and can also induce a variety of genomic rearrangements; they also play an important role in bacterial host specificity and virulence [33, 34]. Transposase IS*200* was found in three high virulent isolated strains encoding the 7 genes (*PmCQ2_1g0197*,* PmCQ2_1g0267, PmCQ2_1g0316, PmCQ2_1g0378, PmCQ2_2g0113, PmCQ2_4g0323,* and* PmCQ2_4g0359*), but IS*200 *was not present in the low virulent strains (PmCQ6, Pm70) or the avirulent strain (Pm3480). The IS*200* elements may adapt to different hosts in closely related genera but stochastic loss can appear in some low virulent or avirulent strains. According to previous reports, IS*200*-related transposons may have already existed in remote stages of bacterial evolution, such as* Salmonellae*, and IS*200*-based methods have been described for the identification of certain* Salmonella *serovars [35]. The function and host range of transposase IS*200* in* P. multocida* still need to be further studied.

*PmCQ2_2g0088* has been suggested to encode a subfamily of ATP-binding cassette (ABC) transporters that have a possible role in remodeling the cell envelope and entry of the pathogen into nonphagocytic cells [36]. Bacterial ABC transporters are essential for the uptake of nutrients, including rare elements such as molybdenum [37]. ABC transporters are integral membrane proteins that actively transport molecules across cell membranes [38], and these three proteins are coded by* modA*,* modB,* and* modC* genes, respectively. The ModA, ModB, and ModC proteins are very similar in various organisms (*Escherichia coli, Haemophilus influenzae, Azotobacter vinelandii, and Rhodobacter capsulatus*) [39]. In this study,* PmCQ2_2g0088* (ModB) is only present in virulent PmCQ2 but absent in PmCQ6.* PmCQ2_2g0088* contains a signal peptide and a* SBP_bac_11* structural domain. The SBP-box gene family is specific to plants and encodes a class of zinc finger-containing transcription factors with a broad range of functions [40]. However, the function of the ModB protein family has not been clearly established;* PmCQ2_2g0088* might affect the virulence of strain and needs to be further studied as a candidate virulence factor.

The present study revealed that* P. multocida* strains carry different virulence genes which may indicate variation in the pathogenicity. It could be speculated that the specific genes of different strains play the most important role for the difference of pathogenicity. By extensive genomics and proteomics analysis, the intensive study on virulence genes provides deeper understanding of host specificity and pathogenesis and also provides insights into the host-microbe interactions and the immunologic mechanism, contributing to the development of novel vaccines.

## Supplementary Material

The detailed information about web links of some databases were shown in the Supplementary Materials, including Nr database, Swiss-Prot database, COG database, IS database and virulence factor database.

## Figures and Tables

**Table 1:** Genome features of sequenced* P. multocida* strains.

Strains	Capsular type	Host	Virulence	Size (Mbp)	Genes	References
PmCQ2	A	Bovine	Highly virulent	2.33	2,002	This study
PmCQ6	A	Bovine	Lowly virulent	2.29	1,970	This study
Pm36950	A	Bovine	Highly virulent	2.35	2,182	[14]
PmHN06	D	Swine	Highly virulent	2.41	2,292	[15]
Pm3480	A	Swine	Avirulent	2.37	2,242	NCBI database
Pm70	F	Avian	Avirulent	2.26	2,090	[8]

**Table 2:** The difference of virulence-associated genes in some or all comparison genomes using BLAST.

Database	PmCQ2	PmCQ6	Pm36950	PmHN06	Pm3480	Pm70	Annotation
Phages-associated genes	PmCQ2_1g0197		Pmu_00900	PMCN06_0843			IS200 transposase
	PmCQ2_1g0267		Pmu_13570				IS200 transposase
	PmCQ2_1g0316		Pmu_13960				IS200 transposase
	PmCQ2_1g0378		Pmu_16290				IS200 transposase
	PmCQ2_2g0113		Pmu_17660				IS200 transposase
	PmCQ2_4g0323		Pmu_18340				IS200 transposase
	PmCQ2_4g0359						IS200 transposase
	PmCQ2_2g0080		Pmu_00570	PMCN06_0130	NT08PM_0122	PM1098	Glycyl-tRNA synthetase subunit alpha
	PmCQ2_2g0148		Pmu_01310	PMCN06_0200	NT08PM_0197	PM1029	Peptidase B
	PmCQ2_2g0349		Pmu_04490	PMCN06_0404	NT08PM_0930	PM0385	Electron transport complex protein RnfC
	PmCQ2_2g0231		Pmu_15590		NT08PM_1618	PM1778	Hypothetical protein
	PmCQ2_6g0020						Hypothetical protein
	PmCQ2_2g0066						Transposase IS605
	PmCQ2_2g0097						Transposase IS605
	PmCQ2_6g0026						Transposase IS605
	PmCQ2_2g0228				NT08PM_0285		Hypothetical protein
	PmCQ2_2g0229				NT08PM_0286		Hypothetical protein
	PmCQ2_2g0230				NT08PM_0288		Hypothetical protein
	PmCQ2_2g0233				NT08PM_0294		Phage major capsid protein
	PmCQ2_2g0234				NT08PM_0295		Hypothetical protein
	PmCQ2_2g0235				NT08PM_0298		Prophage integrase, putative
	PmCQ2_2g0067						Transposase IS200
	PmCQ2_2g0096						Transposase IS200
	PmCQ2_2g0232						Hypothetical protein
	PmCQ2_2g0088						ModB, partial
		PmCQ6_9g0009		PMCN06_2102			Hypothetical protein PMCN06_2102
		PmCQ6_9g0008		PMCN06_2103			Hypothetical protein PMCN06_2103
		PmCQ6_9g0006		PMCN06_2105			Hypothetical protein PMCN06_2105
		PmCQ6_9g0003					Tail assembly protein I
		PmCQ6_9g0001					Host specificity protein, putative, partial
		PmCQ6_9g0005					Tail protein
		PmCQ6_9g0004					Tail assembly protein
Genomic islands	PmCQ2_1g0197		Pmu_00900	PMCN06_0843			IS200 transposase
	PmCQ2_1g0267		Pmu_13570				IS200 transposase
	PmCQ2_1g0316		Pmu_13960				IS200 transposase
	PmCQ2_1g0378		Pmu_16290				IS200 transposase
	PmCQ2_2g0113		Pmu_17660				IS200 transposase
	PmCQ2_4g0323		Pmu_18340				IS200 transposase
	PmCQ2_4g0359						IS200 transposase
	PmCQ2_2g0231		Pmu_15590		NT08PM_1618	PM1778	Hypothetical protein
	PmCQ2_6g0025			PMCN06_0111	NT08PM_0103		Hypothetical protein PMCN06_0111
				PMCN06_1438			
				PMCN06_2110			
	PmCQ2_5g0021			PMCN06_0084	NT08PM_0084		Hypothetical protein PMCN06_0084
				PMCN06_2086			
	PmCQ2_5g0019			PMCN06_0086	NT08PM_0086		Phage terminase, large subunit, pbsx family, putative
				PMCN06_2088			
	PmCQ2_2g0228				NT08PM_0285		Hypothetical protein
	PmCQ2_2g0229				NT08PM_0286		Hypothetical protein
	PmCQ2_2g0230				NT08PM_0288		Hypothetical protein
	PmCQ2_2g0233				NT08PM_0294		Phage major capsid protein
	PmCQ2_2g0234				NT08PM_0295		Hypothetical protein
	PmCQ2_2g0235				NT08PM_0298		Prophage integrase, putative
	PmCQ2_7g0006			PMCN06_0066			Transcriptional regulator
	PmCQ2_5g0013			PMCN06_0092			Hypothetical protein PMCN06_0092
	PmCQ2_5g0025			PMCN06_2080			Hypothetical protein
		PmCQ6_17g0009		PMCN06_0073	NT08PM_0074		Site-specific DNA-methyltransferase (adenine-specific)
				PMCN06_2074			
		PmCQ6_17g0011		PMCN06_0074	NT08PM_0075		Putative bacteriophage protein
				PMCN06_2076			
		PmCQ6_5g0001		PMCN06_0082	NT08PM_0082		Lysozyme
				PMCN06_2084			
		PmCQ6_17g0004		PMCN06_1403			Hypothetical protein
				PMCN06_2067			
		PmCQ6_C4143g0001		PMCN06_1416			Glycoside hydrolase
		PmCQ6_17g0007		PMCN06_2072			Hypothetical protein PMCN06_2072
		PmCQ6_17g0008		PMCN06_2073			Putative replicative DNA helicase
		PmCQ6_17g0010		PMCN06_2075			Hypothetical protein PMCN06_2075
		PmCQ6_9g0009		PMCN06_2102			Hypothetical protein PMCN06_2102
		PmCQ6_9g0008		PMCN06_2103			Hypothetical protein PMCN06_2103
		PmCQ6_9g0006		PMCN06_2105			Hypothetical protein PMCN06_2105

ISs	PmCQ2_1g0197		Pmu_00900	PMCN06_0843			IS200 transposase
	PmCQ2_1g0267		Pmu_13570	PMCN06_0843			IS200 transposase
	PmCQ2_1g0316		Pmu_13960	PMCN06_0843			IS200 transposase
	PmCQ2_1g0378		Pmu_16290	PMCN06_0843			IS200 transposase
	PmCQ2_2g0113		Pmu_17660	PMCN06_0843			IS200 transposase
	PmCQ2_4g0323		Pmu_18340	PMCN06_0843			IS200 transposase
	PmCQ2_4g0359			PMCN06_0843			IS200 transposase
		PmCQ6_12g0001					Putative transposase for insertion sequence IS1162

VFDB	PmCQ2_4g0316		Pmu_13500	PMCN06_1329	NT08PM_1414	PM1994	UDP-3-O-[3-hydroxymyristoyl]; UDP-3-O-acylglucosamine N-acyltransferase
	PmCQ2_1g0631		Pmu_21120	PMCN06_2192	NT08PM_2001	PM1666	Noncanonical purine NTP pyrophosphatase, RdgB/HAM1 family
	PmCQ2_4g0241		Pmu_12710	PMCN06_1257	NT08PM_1342	PM0051	Iron-binding protein FbpA
	PmCQ2_3g0252		Pmu_08050	PMCN06_0796	NT08PM_0537	PM0734	Periplasmic serine protease do/hhoA-like protein
	PmCQ2_2g0162		Pmu_01460	PMCN06_0215	NT08PM_0212	PM1015	Hypothetical protein PM1015
	PmCQ2_1g0106		Pmu_15140	PMCN06_1551	NT08PM_1574	PM1820	Putative virulence effector, SrfC
	PmCQ2_1g0553		Pmu_15880	PMCN06_1607	NT08PM_1650	PM1357	Elongation factor Tu, partial
	PmCQ2_1g0157		Pmu_20230	PMCN06_2025	NT08PM_2100	PM1746	
	PmCQ2_3g0367						Nonspecific tight adherence protein D, partial
		PmCQ6_2g0065	Pmu_09310		NT08PM_0414	PM0846	Nonspecific tight adherence protein D
		PmCQ6_6g0048					Nucleoside-diphosphate sugar epimerase/dehydratase

Secreted proteins	PmCQ2_1g0033		Pmu_14370	PMCN06_1474	NT08PM_1498	PM1897	Hypothetical protein, uncharacterized lipoprotein PM1897
	PmCQ2_3g0252		Pmu_08050	PMCN06_0796	NT08PM_0537	PM0734	Periplasmic serine protease do/hhoA-like protein
	PmCQ2_2g0088						ModB, partial

Membrane proteins	PmCQ2_1g0666		Pmu_21540	PMCN06_2233	NT08PM_2236	PM1230	Penicillin-binding protein 1A
					NT08PM_2237		
	PmCQ2_4g0293		Pmu_13260	PMCN06_1306	NT08PM_1389	PM0004	Bicyclomycin resistance protein-1
		PmCQ6_1g0027	Pmu_21770		NT08PM_2255	PM1212	Mercuric transport protein MerT
		PmCQ6_C4143g0001		PMCN06_1416			Glycoside hydrolase
		PmCQ6_17g0010		PMCN06_2075			Hypothetical protein PMCN06_2075
		PmCQ6_9g0003					Tail assembly protein I
		PmCQ6_14g0071					C4-dicarboxylate ABC transporter permease
		PmCQ6_23g0011					Hypothetical protein, partial

**Table 3:** The distribution of predicted virulence factors among different* P. multocida* strains.

Virulence	Strains	IS 200	PmCQ2_5g0025	PmCQ2_7g0006	PmCQ2_5g0013	IS 605	PmCQ2_2g0088	PmCQ2_3g0367	Capsular type	Host
Highly virulent	PmCQ2	+	+	+	+	+	+	+	A	Bovine
Highly virulent	Pm36950	+	+	+	+	−	−	−	A	Bovine
Highly virulent	PmHN06	+	−	−	−	−	−	−	D	Swine
Lowly virulent	PmCQ6	−	−	−	−	−	−	−	A	Bovine
Avirulent	Pm3480	−	−	−	−	−	−	−	A	Swine
Avirulent	Pm70	−	−	−	−	−	−	−	F	Avian

+ stands for the gene present in certain strain; − stands for the gene absent in certain strain.

## Additional Points

*Availability of Data and Materials*. The genome sequences of PmCQ2 and PmCQ6 have been deposited at GenBank under the accession numbers of LIUN00000000 and LIUO00000000, respectively.
