# Supplementary material for: Comparative Genomics Analysis of Two Different Virulent Bovine Pasteurella multocida Isolates
Source: Int J Genomics. 2016 Dec 14;2016:4512493. doi: 10.1155/2016/4512493 (PMC5192330; doi:10.1155/2016/4512493)
Supplement: Supplementary file 1 — The detailed information about web links of some databases were shown in the Supplementary Materials, including Nr database, Swiss-Prot database, COG database, IS database and virulence factor database. [file 4512493.f1.pdf]

Table S1 The web links of some database in this study

Databases	Web links
Nr database	ftp://ftp.ncbi.nih.gov/blast/db/
Swiss-Prot database	ftp://ftp.uniprot.org/pub/databases/uniprot/
COG database	http://www.ncbi.nlm.nih.gov/COG/
IS database	https://www-is.biotoul.fr
virulence factor database	http://www.mgc.ac.cn/VFs
